# The Role of Effective Communication to Enhance Participation in Screening Mammography: A New Zealand Case

**DOI:** 10.3390/ijerph6020844

**Published:** 2009-02-24

**Authors:** Margaret A Brunton

**Affiliations:** Department of Management & International Business, Massey University, Private Bag 102 904, North Shore Mail Centre, Auckland, 1311, New Zealand; E-Mail: M.A.Brunton@massey.ac.nz; Tel.: +64-9-414-0800 ext 9223; Fax: +64-9-441-8109

**Keywords:** Screening mammography, New Zealand, policy communication

## Abstract

Changes in the organisation of health care have dominated policy initiatives over the past two decades in many countries. An increasing reliance on public health initiatives to prevent or detect disease early has resulted in an increase in programs that screen for cancer in the community. In turn, this accentuates the need to persuasively communicate the value of such initiatives to encourage continued participation. Merely placing screening programs into a community setting is not sufficient to ensure that adequate numbers will voluntarily participate regularly to achieve anticipated cost and mortality savings in the population. In this research the influence of managing communication in a public screening mammography program was investigated. The results revealed that significant opportunities were overlooked for reassurance and information during the physical mammography process. In turn, this highlights the influence of constraints imposed by the structure of the screening program and the resources allocated to the process. This research suggests that it is important to address multiple influences, including ethnic differences, when asking questions about the effectiveness of public health policy, particularly when considering the choices women make about ongoing participation in breast screening programs.

## 1. Introduction

Over the past two decades, an ever-growing demand for improved quality in public health services has driven a comprehensive health sector reform process [1], including an increasing emphasis on public health care initiatives throughout the developed world [2]. The underlying rationale is one of a more efficient and effective use of health resources by providing community interventions which change the way in which health professionals are required to interact with their “customers” [3].

Integral to the increasing reliance on primary health care is the use of technologically-driven health resources which focus on preventing or detecting disease in targeted populations [4]. For example, many countries, including Britain [5,6], America [7,8], Australia [9,10] and New Zealand (NZ) [11], have long advocated participation in screening mammograms which offers the best alternative for reducing mortality from breast cancer in women aged over 50 years [12–15]. As a direct outcome of this rationale, public health policy was introduced to provide a screening mammography program for eligible women in NZ. As ongoing participation is central to successful outcomes, two questions arise about women’s decisions to return regularly for mammograms. First, “What influences shape the way in which staff communicate with women having mammograms?” and second, “What impact did staff communication have on participants in the program?” were the questions which drove the research reported in this paper.

### 1.1. Rationale

Screening for breast cancer is a direct response to the rising incidence of the disease in Western populations internationally, as women in Britain and America experience premature mortality from breast cancer [15,16]. In NZ the disease is the most frequent cause of death from cancer, accounting for some 25% of all cancer registrations for women [17], and in 1998, the NZ government introduced a national screening mammography program providing free two-yearly mammograms for asymptomatic women aged between 50 and 64 years, with eligibility extended to women aged 45–69 years from 2005. It has been estimated that at least 70% of women in the eligible population need to attend regularly for breast cancer screening interventions before either cost or mortality savings are achieved [18,19]. However, some of the above studies have demonstrated that the number of eligible women willing to attend regularly for screening mammograms fails to reach the 70% participation level.

Consistently, ethnic minority groups are significantly less likely to attend regularly for screening mammograms in America [20–22], Britain [23,24] and NZ [25,26]. In NZ there are inequalities in cancer death rates for the indigenous population, with Māori women being 21% more likely to have a diagnosis of breast cancer, and 68% more likely to die of the disease [27]. This reflects overall lower survival rates for the indigenous population [28–32]. Accordingly, as women may choose at any time to opt-out of the program, their experience with the program as a desirable and acceptable option - especially those women of ethnic minority groups who tend to have high mortality rates from advanced disease - matters.

As there is no compulsion to attend, there is now a greater emphasis on the need to understand the potential influences of communication to encourage ongoing participation in screening mammography. Although the importance of identifying and inviting eligible women to be screened has been the focus of international research, recruitment into a screening program is only the beginning of the decision-making process. The effectiveness of any screening mammography program relies on communicating with women not only to encourage them to participate, but also to *continue* to participate. There are a number of decision points about ongoing participation; thus the need for health personnel to understand the potential influence of effectively managing the process *during* mammography underlies questions about the influences that the reform-driven structural environment of the public health service has on their performance and the subsequent experience and perceptions of participants in the program.

### 1.2. The Role of Communication

Certain health communication models have developed as a result of this transition period in the health sector, and a number of different models such as health promotion, disease prevention, disease detection and social marketing are used to attempt to encourage individuals in a “well” population to participate in community screening programs. The study of communication in screening mammography programs suggests that there is no single, categorical means of effective communication. There are three distinct phases in the mammography process, and women require different types of communication to allow them to make informed decisions at each stage of the process [33,34]: the invitation to participate initially, the encouragement to return for repeat mammograms, and the communication of results (including untoward findings such as false positives) [35]. This project is concerned only with the second stage of the process (encouraging women to return for repeat screening), the other stages having been far more widely researched.

The style of communication that is dominant in the three phases of communicating screening mammography may be further defined by a definition of language which distinguishes between *transactional* communication that expresses “content”, and *interactional* communication that expresses “social relations and personal attitudes” [36]. Although the demarcation is not always complete, the dichotomy provided a useful distinction for this research. The first phase of communication to encourage women to participate in screening mammography is based primarily on print resources, such as pamphlets, posters and letters of invitation. These resources use transactional language to convey factual information. The function of transactional language is to transfer information efficiently, which in this case, is integral to providing the resources for facilitating the participation of eligible women in the breast screening program. Although there is typically some interactional communication between women considering breast screening and health professionals in the community, interactional communication primarily occurs when women are interacting with the staff during the physical process of mammography. Accordingly, the second phase of communication in screening mammography is centered on interactional language, characterized by the interpersonal domain [36].

The role of effective communication in identifying and inviting eligible women to be screened has been the focus of ongoing international research, which has reflected elements of both transactional and interactional communication. The role of letters of invitation [37–42], phone calls [43,44], general practitioners [41,45], and personal contact programs [46,47] has been examined exhaustively; however, there has been less focus on how communication may influence subsequent decisions to return.

### 1.3. Decisions to Return

As there are a number of decision points about ongoing participation, women’s experience of breast screening will not only strongly influence decisions they make about re-attendance [48,49], but also encourage those women to influence others in their social network through discussions about the experience [50]. There are risks and limitations [51], and concerns about exposure to radiation [52]. The procedure itself tends to be overlain with anxiety [53,54], and in NZ, women primarily attend for reassurance [50,55]. Research with a communication focus has argued for the importance of clear and simple information about the procedure to increase acceptability for women [56,57], by reducing the consistently high levels of anxiety that women appear to experience when undergoing screening mammography [57]. The importance of facilitating questioning in a supportive environment has also been found to be integral to a more acceptable process for women, and will influence their decisions to return for further mammograms [58].

Accordingly, it is important to understand whether decisions being made in a screening program are effectively dealing with potential barriers to regular participation. If a program has been resourced and offered to the community it needs to be successful in attaining the participation rate required to achieve effective public health goals. This research addresses questions about possible influences on encouraging eligible women to regularly participate in a regional population-based breast screening program in NZ. The two questions which drove this research were first, “What influences shaped the way in which staff communicated with women having mammograms?” and second, “What impact did staff communication have on participants in the program?”

## 2. Method

In this paper, the responses of women who were recruited into a regional screening mammography program are considered, as well as those of experienced health professionals providing the service. As there has been considerable research about encouraging women to enroll in screening programs, it was of particular interest to this research to examine what influences shaped the way in which staff communicated with women having mammograms, and what impact the process had on participants.

Questionnaires, focus groups and interviews were used for data collection. The self-completion questionnaire survey included questions related to the interactive communication environment to inform, educate and alleviate anxiety during the physical process of mammography. At the time of the study, the organisation comprised 19 female staff, of which 13 worked in the breast screening unit, carrying out the physical process of mammography in a regional program. Four women were employed as community health educators to distribute resources to general practitioners and hold education sessions to encourage the eligible population to take part in the breast screening program, and the remaining respondent, the manager, was responsible for the overall coordination of the service.

The data for this case were collected through semi-structured interviews with all 19 staff members responsible for providing the screening mammography program. The interviews were undertaken to elicit individual accounts, including various aspects of the way in which staff interpreted and responded to their environment. The interviews lasted for between 90 to 120 minutes and all interview notes were fully transcribed. Patterns were identified in order to identify narrative themes relevant to the research question to address RQ1: “What influences shaped the way in which staff communicated with women having mammograms?”

The questionnaire survey included both open and closed questions relating to women’s experience of breast screening. The questions were generated from a review of international literature to identify how women responded to communication from the breast screening program both to encourage them to participate in, and return for, regular screening mammography. Following ethical approval a two-stage pre-test was conducted among seven groups of a total of 60 women. The survey was sent to a random sample of 1085 women, drawn from the 14,000+ women on the screening database. As both confidentiality and anonymity were ethical prerequisites of the study, any access to women’s details was precluded to an outside researcher, and the sample selection could be obtained only by request. Requested variables such as prior experience of screening or family history were not available from the database.

An invitation to participate in a focus group interview was included with the survey, and 44 women took part in group interviews, lasting for between 60 and 90 minutes. The responses to the questionnaire and focus group interviews were used to address RQ2: “What impact did staff communication have on the participants in the program?”

### 2.1. Study Population

In the initial mailing group of 1,085, 13 people were found to be either not available or not suitable and were removed from the list (eight deceased, four living overseas, one male). Combined with 21 GNA (gone, no address) returns, the net number of questionnaires distributed was 1051. Altogether, 629 (61%) questionnaires were returned from respondents, of which 611 (58%) were completed. Incomplete questionnaires were excluded from analysis. Subsequently, the “other” ethnic population of 15 women was excluded from statistical analysis because of the small number in this category, which left a total of 596 respondents, as described in Table 1 below.

### 2.2. Analysis

The data from the survey questionnaire were analyzed using SPSS. Chi-squared (χ^2^) tests for independence in contingency tables were used to identify trends and assess the significance of associations between demographic characteristics and other variables. All comments from the questionnaires, focus groups and interviews were fully transcribed. The majority of questions in the survey were directive or closed, including those which used a Likert scale that related to the program interface. Closed questions allowed women to respond to a series of tick boxes. Open or non-directive questions were asked when it was believed to be important that women could describe various outcomes, for example, perceived information deficits. Each section of the questionnaire provided opportunities for comments, and 1248 confidential comments, which comprised several pages of open and detailed feedback, were generated.

Qualitative analysis of written comments and staff interviews relied on content analysis [59] of the comments generated by survey and focus group respondents, and also those from the interviews held with staff members in a screening mammography program. First, to ensure familiarity with the material, the transcribed interview data and comments from the questionnaire survey were read on several occasions over a number of weeks. Initially, the data were analysed for frequently occurring descriptors, and were collated to develop consistent categories or themes using thematic analysis [60] to identify underlying narratives which are recurrent, repetitive and emphasised by vocal inflection or non verbal cues (noted in the questionnaire data by exclamation marks or capital letters, and by vocal inflection or volume in spoken dialogue). Repetitiveness and recurrence were identified after several readings of the data to identify those themes relevant to the research questions. For example, in this study, although the word “anxious” was used repetitively as a descriptor, associated words such as, “worried”, “stressed”, “upset” and “frightened” were also used to describe the type of anxiety experienced by respondents; therefore these words were identified as recurrent descriptors of anxiety. An independent researcher was briefed on the study and carried out an analysis of the data using the same criteria, with a high level (92%) of intercoder agreement. To ensure that the interpretation of the material was credible to participants, secondary interviews were subsequently held with available participants.

## 3. Results

Women in this research were asked about their experiences, including whether they believed they had received clear explanations at each stage in the breast screening process, identified as central to an acceptable process for women, as suggested earlier [56,57]. Their responses are illustrated in Table 3.

Although overall 60% stated that they had received clear explanations, differences were evident as the Māori and Pacific women, ethnic minority groups in this research, consistently demonstrated different information needs, reporting difficulty with the introductory verbal information and the way in which it was explained. Pacific (49%) and Māori (62%) women were less likely to state they had received a clear explanation of the procedure than European (70%) or Asian women (74%, p=0.009) Similarly, Pacific (34%) and Māori (58%) women were less likely (p =0.001) to report receiving a clear explanation about the test results than either European (64%) or Asian (64%) women.

As one Pacific woman wrote in the questionnaire, “English is my second language and staff mostly took things for granted when it came to explanation,” which she reported made her feel reluctant to ask further questions. In the focus groups respondents commented that while the procedures may have been explained to them, they did not feel able to respond. For example, Māori women described spoken information as “confusing”, “too fast”, “overwhelming”, or complained of not receiving any. Others detailed how they felt “there was no room for questions”, and more importantly, as one Māori woman explained, “Staff took the silence to mean that I understood what was being said when I did not respond, and continued [with the process].”

### 3.1. The Role of Interactive Questioning

To further explore whether actively participating in the communication process through questioning the process would enhance the screening experience for participants [58], responses from women about the perceived clarity of explanations were analyzed to see if they were related to the comfort level about asking questions. In other words, respondents were given the time and opportunity to question whether the nature of the explanations was relevant to their perceptions of an acceptable mammography experience. In response to whether women felt comfortable asking questions during their mammogram, the results illustrate that the perceived clarity of each of the above sources of information was significantly related to the comfort level respondents felt about asking staff questions, as outlined in Table 4.

Overall, 44% of women “always” felt comfortable asking questions, 28% “usually” and 17% “sometimes” felt comfortable about asking questions. The remaining 11% did not feel they could ever question, explaining their perceptions of a “rushed” and “clinical” environment. To further assess the process, the explanations received were again analyzed to see if they were related to the comfort level about asking questions. The women who were most comfortable about questioning ultimately believed they had received clear explanations more often than those who did not.

There were no significant differences among age groups; however, differences were evident among ethnic groups. NZ European women appeared to be the most comfortable asking questions with 82% answering “always” or “usually” compared with Māori (64%), Pacific (39%) and Asian women (60%). Pacific women were the most reluctant (p<0.001) to question, with 61% feeling comfortable about asking “sometimes” or “never” (M=36%, NZE=18%, A=40%).

#### The influence of ethnicity

When asked to clarify the difficulties they experienced, several Māori women commented in the focus groups that while the procedures were explained to them, they often felt there were few opportunities to interact with staff, which perhaps explains why many Māori and Pacific women described their mammograms as “lonely and isolating” and “unpleasant”. As one respondent explained, “I thought it was impersonal actually, to me it took over. It was a lonely, scary, isolating experience. They just put you in there waiting your turn. And then they use the big words – I don’t know what they mean.” Pacific women said they “tended to nod and say ‘yes’ because they didn’t want to offend”. As one respondent explained, “Because we just say ‘yep, yep’, especially our old people, and really we don’t know what they (the staff) are saying.”

#### The influence of anxiety

The process of breast screening is associated with varying levels of anxiety for women [53] with 77% of respondents reporting some level of worry about getting breast cancer as the most prevalent source of anxiety. As those women who felt “quite” or “very” worried (28%) about getting breast cancer were less likely to report feeling comfortable about asking questions than those who were only “a bit” or “not” worried (72%, p<0.001), anxiety appears to directly influence the communication process. Pacific (59%) and Māori (35%) women experienced significantly higher levels of feeling “very worried” about breast cancer than NZ European (20%), or Asian (20%) women (p<0.001).

#### Future decisions about participation

As women are required to make ongoing decisions about whether they will continue to receive regular mammograms their future intentions are important. As it is possible for women at any stage to choose to select themselves out of the program, respondents were asked about their intentions for future participation in mammography. In this case, the study comprised a sample of respondents of whom 70% had two or more mammograms. They were also a highly committed sample of women who were very supportive of the need for screening. The majority of respondents viewed breast screening as very important (88%) or important (11%). However, some were reconsidering future attendance. For the 44 women who reported this decision, they were significantly more likely to do so after only one mammogram (p<0.001). Of the 104 women who have had one mammogram, 14% had either made, or were considering, a decision not to return. Among the 204 respondents who have had two mammograms, 9% had decided similarly. Of the 303 respondents who had experienced three or more mammograms, 4% were either undecided, or had made a decision not to continue. The three primary reasons given for the decision were pain, anxiety and lack of time.

### 3.2. Staff Responses to the Data

As communication is a two-way process, the semi-structured voluntary interviews with staff members yielded an interesting perspective on the intersection of multiple discourses and the implications for performance outcomes.

#### Striving for effectiveness and efficiency

Enhancing quality through greater efficiency in the health service was prevalent, for example, making sure that a certain number of women were screened during a specified period of time. Other considerations appeared to be subservient as staff related how it was “essential to reach financial targets” and necessary to “work to “justify the expense” of the program, which was “not always easy”. The emphasis on economic rationalism appeared to result in a service legitimated through accountability by numerical performance targets. However, such a focus was problematic as staff were allocated a certain period of time to conduct mammograms. With the aging baby boom cohort, increasing numbers of women were eligible to participate, and the service was facing the reality of screening growing numbers of women within the same available time frame, using the same resources. Staff often related that they were “expected to make things work”. The subsequent influence on the values transferred to the interaction between the staff and participants in a time and resource constrained process.

#### Performance measures

Performance targets have been introduced into the health sector through initiatives to ensure such targets are met and organisations providing services to the national breast screening program are evaluated through audits carried out by an external monitoring committee, which regularly assesses and compares the performance of organisations under contract to the government-funded program. It is no longer possible simply to provide a service without paying attention to the cost, without accountability being measured in expenditure. Once again, the service was required to reach economic utility. In the words of the manager: “We must complete the round [the period allocated to screen all eligible women in a given population area] in time.” The resulting imperatives subjugated the needs of participants to the exigency of meeting targets. As previously debated, an ascendant need for organisational efficiency over the service ethic requires changes in behaviour, and in turn, this may inadvertently cause harm [61].

In this research, staff strove to provide “excellence in everything we do”, although as one explained, “Sometimes it’s so hard just keeping your focus on giving your lady the best you can”. Demands for enhanced performance and service quality need to be adjusted and readjusted to organisational realities [62]. However, in this case, the ongoing waves of reform in the NZ health sector, recognised as the most comprehensive and radical in the OECD [63,64] meant that the force of change demanded adjustment and readjustment from those who were providing the service and the result was described by a senior staff member as “a high level of burnout among the staff that is a constant worry”.

#### Customer orientation

In this case, the structural influences of both monitoring and performance targets affected the way in which staff communicated with women taking part. “In constructing the organisation as one thing as opposed to another, certain lines of action are invited and others discouraged”. Control was perceived as necessary by staff because of the pace of appointments that precluded the prospect of spending longer with women, as the emphasis was on keeping the momentum going. Similarly, if women exhibited anxiety in the unfamiliar environment, some conflict was reported by staff as they coped with the dilemma of the need for efficiency. When discussing the apparent desire of some women to talk about the process when being screened, one staff member explained that trying to incorporate questions and explanations into the tight time frame “can be an issue”. If women are going to be willing to participate in an anxiety-inducing program such as screening for breast cancer, a supportive environment and communication are integral to all stages of the process [56,57].

The ongoing tendency for both Māori and Pacific women to present later with advanced disease and experience higher mortality rates in NZ [25,30–32] reflects an international trend, and is thus a compelling reason to address this issue. Māori health educators expressed their opinion that there had been a lack of consultation with indigenous Māori. As one explained, the right to consultation provided under the Treaty of Waitangi was overlooked: “Although we had a pilot program and had the opportunity to make changes for Māori, there was never any input allowed from us at management level,” which is antipathy to Māori protocol. The outcome was that Māori and Pacific health educators perceived an “inflexibility” of the program in accommodating the needs of their women. They further expressed an ongoing desire to move away from “the monocultural way of mainstream” in recognition of “another way” to approach screening within parameters that were acceptable to all parties.

### 3.3. Limitations

Admittedly, although obtaining a random sample of women from the breast screening database provided a sample of women who had been exposed to the communication from the breast screening program, it also eliminated those who had not attended. Additional bias resulted because the respondents were self-selecting. Overall, population-based screening programs provide a service to an eligible population that is largely self-selected. To account for the non-response bias, surveys returned after a reminder letter was sent were coded as “late returns” and responses from this group compared with earlier returns. There were no significant differences among variables between respondents, which suggests that the sample was likely to be representative of the remaining population of non-responders in this study.

## 4. Discussion

The ethnic diversity of the population has demonstrated significant differences in responses to communication during screening mammography. Māori and Pacific women appeared to need a more personal setting than Asian and NZ European women in this study. Although restraint is necessary in generalizing across geographical boundaries, minority groups in America and Britain are also more likely to report feeling confused and having unmet needs for clear information about breast screening than mainstream populations [66,67]. Such findings highlight both the education [68] and language needs of minority groups that may not “fit” the prevailing approach [69]. As Māori and Pacific women expressed shyness and reluctance to question, the experience of breast screening is one that appears to compound their already high anxiety level.

The contrasting experiences and perceptions of ethnic groups of women who chose to participate in screening mammography were also reflected in the level of anxiety experienced. In this study, women who were more anxious were less likely to actively question health professionals, which suggests “defensive avoidance” towards threatening information [70]. For example, reluctance to address anxiety-provoking threats to health was prevalent in an earlier study of women’s attitudes towards breast self-examination [71]. Also, the highest screening attendance in screening programs occurred with those women who were “a bit worried”, in contrast to those women who were “very worried” about the possibility of breast cancer [6]. Since the communication strategies used by health care professionals play a key role in determining health outcomes [72], it appears to be important to frame communication within a supportive environment. The emphasis on encouraging discussion needs to move beyond the transactional frame, and recognize the potency of interactional communication in addressing anxiety in participants.

As the emphasis on greater effectiveness and efficiency dominates the health sector, questions about the experience of those invited into screening programs are vitally important. This paper provides insight into the other side of the experience: those who are providing the service. As the progressive ageing of the baby boom cohort means that large numbers of women are now becoming eligible to participate in the program, the service is facing the reality of screening increasing numbers of women. As performance targets drive the service, staff are faced with providing an acceptable, accessible and efficient service. The combination of the questionnaire survey and individual and focus group interviews provided data that clearly indicated that the environment served to undermine communication from staff in the breast screening program with their target audience. The powerful rhetoric of economic rationality influence activities of those in the health service [65], and in this case, served to displace the reported effort to attain a “woman-centered” service as staff perceived the ascendancy of minimal time to provide the service.

In contrast, it was noticeable that the outcome did not sit comfortably with women who participated in the program. From the open and detailed feedback in the survey, women having mammograms did challenge the authority of economic rationality, which has “subsume[d] many political and social languages” in order to redefine everything in “purely economic terms” [73]. The emphasis on an ideological investment of efficiency to enhance the quality of the service was not readily accepted. However, participants reported a reluctance to complain “in case the service gets taken away” – thus the voice of the customer was relatively absent.

As there has been no particular approach that has consistently identified why women choose to resist regular participation in breast screening, many important and unresolved questions remain. Every attempt needs to be made to ensure that accurate, comprehensible communication is available; however the potential anxiety associated with participation in screening programs for breast cancer must also be recognized. The management of effective communication was emphasised in this research as the opportunity to question is an important variable in education and information transfer in a breast screening environment. As cultural differences and increased levels of anxiety may underpin a reluctance to have a mammogram in some groups of women, encouragement to question and interact with staff appears integral to an acceptable process.

## 5. Conclusions

Although this study was limited to one organisation within the health sector, it has provided insight into the paradoxes that exist within the need to provide a quality screening program by improved effectiveness (through meeting desired outcomes for the community and removing potential barriers to participation) and efficiency (through cost savings). To try to argue against the desirability of the most efficient and effective outcomes for public health services would be foolhardy, though it appears that an emphasis on efficiency may serve to impound the equally critical need for effectiveness. Certainly it is problematic to attempt to measure “effectiveness” compared to “efficiency” when assessing the benefit of a public health service [74]. On the other hand, if government intentionally sets out to restructure the health care system with an overriding objective of improving quality through enhanced efficiency and effectiveness, both are equally important.

So why does it all matter? This project, if anything, highlights the complexity of work life in health care organisations that are in a constant flux of adaptation. The grass-roots reality is that health professionals are all busy simply trying to get the job done. They are facing difficulties and heavy workloads as they actively engage in attempts to meet criteria which demand high levels of efficient performance and responsiveness to the needs of service users. Distinctions in the ideological perspectives of efficiency that were designed and legitimated to displace the service ethic as a distinctively human activity were transposed into the screening process as the service adapted to the tensions and contradictions of the organisational imperative. For staff, it appeared that the distinction between the rhetoric and the reality created a paradox as they encountered the need for transactional efficiency which collided with the interactional communication needs of service users.

If accountability is measured through numbers of inputs and outputs to gauge efficiency, the critical nature of an acceptable experience for service users becomes overlooked. If the service fails to take into account the needs of voluntary participants, they are unlikely to return regularly. In this case, if women are not prepared to participate regularly in the screening mammography program, it cannot be considered effective, regardless of how efficiently the service is run. For individuals to accept the possibility of disease requires a measure of persuasion, and thus emphasises both the need for partnership and effective communication. Ultimately, some women may choose to respond to invitations to participate in breast screening with an ultimate decision of “no thank you”. That is the way things sometimes work. Nonetheless, if public policy results in ineffective services whole populations run the risk of diminished health. The practical and moral imperatives in this case are clear.

## Figures and Tables

**Table 1.** Stages of data collection.

Stage 1	Self-completion questionnaire survey of participants
	Follow-up letter sent within 6 weeks (4 months)
	Data entered into SPSS and content analysis of qualitative data to identify common categories (2 months)
Stage 2	Focus groups with participants to obtain information and seek feedback on data (3 months)
Stage 3	Semi-structured interviews with staff (3 weeks)
	Transcription and content analysis of interview data (3 months)
	Secondary interviews with staff to obtain feedback (2 weeks)

**Table 2.** Demographic information obtained from respondents.

**Age**		**Domicile**	
50 – 54	227	Live in a city	190
55 – 59	207	Live in a rural town	318
60 – 64	162	Live in the country	88
**Ethnic Origin**		**Occupation**	
Māori	155	Wages or salary	272
European	348	Unpaid work in home	125
Pacific	51	Self employed	72
Asian	42	Retired	127
**Level of Education**		**Annual Income**	
Primary School	29	Less than $15,000	142
Secondary School	370	15,000 to $30,000	150
University	69	30,001 to $50,000	101
Trade or Polytech	48	Greater than $50,000	63
Other sources	80	Don’t wish to answer	140

**Table 3.** Clear explanations received about the screening process.

Received a clear explanation of what breast screening is looking for	65%
Received a clear explanation of the breast screening procedure	66%
Received a clear explanation of when and how test results will be made available	62%
Received a clear explanation of the test results	60%
Received a clear explanation of any further action required	47%

**Table 4.** Comfort level of asking staff questions compared with clear explanations received crosstabulation.

	Comfort level asking staff questions		
Source of information	Always/usually	Sometimes/never	Significance level
Received a clear explanation of what breast screening is looking for	78%	22%	p=0.000
Received a clear explanation of the breast screening procedure	79%	21%	p=0.000
Received a clear explanation of when and how test results will be made available	78%	22%	p=0.000
Received a clear explanation of the test results	77%	23%	p=0.001
Received a clear explanation of any further action required	78%	22%	p=0.002

## References

[1] Thynne I (2003). Making Sense of Public Management Reform: ‘Drivers’ and ‘Supporters’ in Comparative Perspective. Public Manag. Rev.

[2] MacDougall C (2001). Thoughts on Barriers and Enablers for Incorporating Ordinary Theorizing into the Community Participation in Health Debate. Aust. Health Rev.

[3] Cheney G, Zorn T (1999). Is Serving a Customer a Noble Mission or Misguided Ideology?. At Work.

[4] Tabar L, Yen MF, Vitak B, Chen HT, Smith RA, Duffy SW (2003). Mammography Service Screening and Mortality in Breast Cancer Patients: 20-Year Follow-Up Before and after Introduction of Screening. Lancet.

[5] Greenwood-Haigh L Mammographic Surveillance in the Follow up of Early Primary Breast Cancer in England: A Cross-sectional Survey Radiography 2008 In press, doi:10.1016/j.radi.2008.08.005

[6] Sutton S, Bickler G, Sancho-Aldridge J, Saidi G (1994). Prospective Study of Predictors of Attendance for Breast Screening in Inner London. J. Epidemiol. Community Health.

[7] Walter LC, Lewis CL, Barton MB (2005). Screening for Colorectal, Breast, and Cervical Cancer in the Elderly: A Review of the Evidence. Am. J. Med.

[8] McBride CM, Curry SJ, Taplin S, Anderman C, Grothaus L (1993). Exploring Environmental Barriers to Participation In Mammography Screening in an HMO. Cancer Epidemiol. Biomarker. Prev.

[9] Taylor R, Morrell S, Estoesta J, Brassil A (2004). Mammography Screening and Breast Cancer Mortality in New South Wales, Australia. Cancer Cause. Control.

[10] Turnbull D, Irwig L, Simpson JM, Donnelly N, Mock P (1995). A Prospective Cohort Study Investigating Psychosocial Predictors of Attendance at a Mobile Breast Screening Service. Aust. J. Public Health.

[11] Armstrong W, Borman B (1996). Breast Cancer in New Zealand: Trends, Patterns, and Data Quality. N ZMed. J.

[12] Allgood PC, Warwick J, Warren RM, Day NE, Duffy SW (2008). A Case-Control Study of the Impact of the East Anglian Breast Screening Programme on Breast Cancer Mortality. Br. J. Cancer.

[13] Freedman DA, Petitti DB, Robins JM (2004). On The Efficacy of Screening for Breast Cancer. Int. J. Epidemiol.

[14] Haynes M, Smedley B (1999). The Unequal Burden of Cancer: An Assessment of NIH Research and Programs for Ethnic Minorities and the Medically Underserved.

[15] Miettinen OS, Henschke CI, Pasmantier MW, Smith JP, Libby DM, Yankelevitz DF (2002). Mammographic Screening: No Reliable Supporting Evidence?. Lancet.

[16] Evans D, Lalloo F, Shenton A, Boggis C, Howell A (2001). Uptake of screening and prevention in women at very high risk of breast cancer. Lancet.

[17] NZHIS (2005). Cancer: New Registrations and Deaths 2001.

[18] Austoker J, Fagge D, Gray S, Patnick J (1995). Messages about Breast Screening: Guidelines for Health Promotion Specialists, Primary Care Teams and Screening Unit Staff.

[19] De Koning HJ (1996). Is Mass Screening for Breast Cancer Cost-Effective?. Eur. J. Cancer.

[20] Denhaerynck K, Lasaffre E, Baele J, Cortebeeck K, van Overstraete E, Buntinx F (2003). Mammography Screening Attendance: Meta-Analysis Of The Effect Of Direct-Contact Invitation. Am. J. Prev. Med.

[21] Giuliano A, Papenfuss M, de Zapien J, Tilousi S, Nuvayestewa L (1998). Breast Cancer Screening among Southwest American Indian Women Living on Reservations. Prev. Med.

[22] Selvin E, Brett KM (2003). Breast and Cervical Cancer Screening: Sociodemographic Predictors among White, Black, and Hispanic Women. Am. J. Public Health.

[23] Kernohan E (1996). E.M. Evaluation of a Pilot Study for Breast and Cervical Cancer Screening with Bradford’s Minority Ethnic Women; A Community Development Approach, 1991–93. Brit. J. Cancer.

[24] Pfeffer N, Moynihan C (1996). Ethnicity and Health Beliefs with Respect to Cancer: A Critical Review of Methodology. Brit. J. Cancer.

[25] Lethaby AE, Mason BH, Holdaway IM, Kay RG (1992). Age and Ethnicity as Prognostic Factors Influencing Overall Survival in Breast Cancer Patients in the Auckland Region. N Z Med. J.

[26] Mason BH, Holdaway IM, Newman PD, Kay RG, Benjamin C, Harvey J (1994). Treatment of Breast Cancer in the Auckland Region 1976–85. N Z Med. J.

[27] Robson B, Purdie G, Cormack D (2006). Unequal Impact: Māori and Non-Māori Cancer Statistics 1996–2001.

[28] Ajwani S, Blakely T, Robson B, Tobias M, Bonne M (2003). Decades of Disparity: Ethnic Mortality Trends in New Zealand 1980–1999.

[29] Blakely T, Ajwani S, Robson B, Tobias M, Bonne M (2004). Decades of Disparity: Widening Ethnic Mortality Gaps from 1980 to 1999. N Z Med. J.

[30] Gill A, Martin I (2002). Survival from Upper Gastrointestinal Cancer in New Zealand: The Effect of Distance from a Major Hospital, Socioeconomic Status, Ethnicity, Age and Gender. ANZ J. Surg.

[31] Cormack D, Robson B, Purdie G, Ratima M, Brown R (2005). Access to Cancer Services for Māori.

[32] Jeffreys M, Stevanovic V, Tobias M, Lewis C, Ellison-Loschmann L, Pearce N, Blakely T (2005). Ethnic Inequalities in Cancer Survival in New Zealand: Linkage Study. Am. J. Public Health.

[33] Bonevski B Perkins J Sanson-Fisher R Lightfoot J The Effectiveness of Recruitment Strategies to Mammographic Screening: A Review of the Literature (1989–1996) Report prepared for BreastScreen NSW: Cancer Education Research Program (CERP), 1996

[34] Gladding B “Where Women Are”. Health Education, Promotion, and Information Options for the National Breast Cancer Screening Programme in New Zealand. Unpublished report to the Joint RHA Public Health Group. Lower Hutt Plus Ultra 1997

[35] Austoker J, Ong G (1994). Written Information Needs of Women who are Recalled for Further Investigation of Breast Screening: Results of a Multicentre Study. J. Med Screening.

[36] Brown G, Yule G (1983). Discourse Analysis.

[37] Mandelblatt J, Kanetsky PA (1995). Effectiveness of Interventions to Enhance Physician Screening for Breast Cancer. J. Fam. Pract.

[38] Rimer BK (1992). Understanding the Acceptance of Mammography by Women. Ann. Behl. Med.

[39] Sienko DG, Hahn RA, Mills EM, Yoon-DeLong V, Ciesielski CA, Williamson G, Teutsch SM, Klenn PJ, Berkelman RL (1993). Mammography Use and Outcomes in a Community. Cancer.

[40] Miller D, McNoe B, Elwood M, Doyle TC (1998). The General Practitioner’s Role in Breast Cancer Screening: A Survey in Otago-Southland. N Z Med. J.

[41] Richardson A, Williams S, Elwood M, Bahr M, Medlicott T (1994). Participation in Breast Cancer Screening: Randomised Controlled Trials of Doctors’ Letters and of Telephone Reminders. Aust. J. Public Health.

[42] Brunton M, Thomas D (2002). Privacy or Life: How do Women find out about Breast Screening Services?. N Z Med. J.

[43] Taplin SH, Barlow WE, Ludman E, MacLehos R, Meyer DM, Seger D, Chin C, Curry S (2000). Testing Reminder and Motivational Telephone Calls to Increase Breast Screening: A Randomized Study. J. Natl. Cancer Inst.

[44] Saywell RM, Champion VL, Skinner CS, McQuillen D, Martin D, Maraj M (1999). Cost-Effectiveness Comparison of Five Interventions to Increase Mammography Screening. Prev. Med.

[45] Phillips KA, Kerlikowske K, Baker LC, Chang SW, Brown ML (1998). Factors Associated with Women’s Adherence to Mammography Screening Guidelines. HSR: Health Serv. Res.

[46] Skinner CS, Strecher VJ, Hospers H (1994). Physicians’ Recommendations for Mammography: Do Tailored Messages make a Difference?. Am. J. Public Health.

[47] Calle EE, Moss RE, Miracle-McMahill HL, Heath CW (1994). Personal Contact from Friends to Increase Mammography Usage. Am. J. Prev. Med.

[48] Dolan NC, Feinglass J, Priyanath A, Haviley C, Sorensen A, Venta LA (2001). Measuring Satisfaction With Mammography Results Reporting. J. Gen. Intern. Med.

[49] Elwood M, McNoe B, Smith T, Bandaranayake M, Doyle TCA (1998). Once Is Enough - Why Some Women Do Not Continue To Participate In A Breast Cancer Screening Programme. N Z Med. J.

[50] McNoe B, Richardson AK, Elwood JM (1996). Factors Affecting Participation in Mammography Screening. N Z Med. J.

[51] Wilson RM (2000). Screening for Breast and Cervical Cancer as a Common Cause for Litigation. Br. Med. J.

[52] Bakker DA, Lightfoot NE, Steggles S, Jackson C (1998). The Experience and Satisfaction of Women Attending Breast Cancer Screening. Oncol. Nurs. Forum.

[53] Brunton M, Jordan C, Campbell I (2005). Anxiety Before, During, and After Participation in a Population-Based Screening Mammography Programme in Waikato Province, New Zealand. N Z Med. J.

[54] Lightfoot N, Steggles S, Conlon M, Darlington G, Bakker D, Jackson C (1996). Factors Associated With Mammographic Discomfort in Breast Screening Programme Participants. Curr. Oncol.

[55] Richardson A Evaluation of a Pilot Breast Cancer Screening Programme Unpublished doctoral dissertation, University of Otago, Dunedin, New Zealand, 1996

[56] Marteau TM (1990). Reducing the Psychological Costs. Br. Med. J.

[57] Marteau TM, Kidd J, Cuddeford L (1996). Reducing Anxiety in Women Referred for Colposcopy using an Information Booklet. Br. J. Health Psychol.

[58] Kamm B (2000). Communicating With Mammography Patients. Radiol. Technol.

[59] Krippendorff K (2004). Content Analysis: An Introduction to its Methodology.

[60] Owen WF (1984). Interpretive Themes in Relational Communication. Quarterly Journal of Speech.

[61] Plant R, Plant R, Beech M, Hickson K (2004). Ends, Means and Political Identity. The Struggle for Labour’s Soul.

[62] Reed M, Kirkpatrick I, Lucio M (1995). Managing Quality and Organizational Politics: TQM as a Governmental Technology. The Politics of Quality in the Public Sector.

[63] French S, Old A, Healy J (2001). Health Care Systems in Transition: New Zealand.

[64] Pollitt C, Bouckaert G (2004). Public Management Reform: A Comparative Analysis.

[65] De Cock C Organisational Change and Discourse: Hegemony, Resistance and Reconstitution Management 1998 1 1 22 (p.2).

[66] Meissner HI, Rimer BK, Davis WW, Eisner EJ, Siegler IC (2003). Another Round in the Mammography Controversy. J. Womens Health.

[67] Morton E, Tamobor E, Rimer BK, Tessaro I, Farrell D, Siegler IC (1996). Impact of National Cancer Institute’s Revised Mammography Screening Guidelines on Women. Women’s Health Issue.

[68] Croft E, Barratt A, Butow P (2002). Information about Tests for Breast Cancer: What are we Telling People?. J. Fam. Pract.

[69] Woloshin S, Schwartz L, Katz S, Welch G (1997). Is Language a Barrier to the Use of Preventive Services?. J. Gen. Intern. Med.

[70] Millar MG Millar K The Effects of Anxiety on Response Times to Disease Detection and Health Promotion Behaviors J. Behav. Med 1996 194 401 413 (p.411). 8836829 10.1007/BF01904765

[71] Meyerowitz BE, Chaiken S (1987). The Effect of Message Framing on Breast Self-Examination Attitudes, Intentions, and Behavior. J. Pers. Soc. Psychol.

[72] Kreps GL, O’Hair D, Hart MC, Kreps GL, O’Hair D (1995). Communication and Health. Communication and Health Outcomes.

[73] Cheney G ‘It’s the Economy, Stupid!’ Aust. J. Commun 1998 253 25 44 (p.31).

[74] Esmail N Walker M Yeudall S How good is Canadian Health Care? 2004 Report. An International Comparison of Health Care Systems The Fraser Institute Vancouver 2004 Available at http://www.fraserinstitute.org/commerce.web/product_files/HowGoodCanadianHealthcare2004.pdf (Accessed February 2009)

